# Surgical intervention of myocardial bridge combined coronary artery disease: could a combination of supra-arterial myotomy and CABG be a better option?

**DOI:** 10.1186/s13019-023-02251-z

**Published:** 2023-04-10

**Authors:** Xi-Ruo Xu, Ming-Kui Zhang, Qing-Yu Wu, Li-Xin Fan, Hui Xue

**Affiliations:** Heart Center, First Hospital of Tsinghua University, No. 6 1st Street, Jiuxianqiao, Chaoyang District, Beijing, 100016 China

**Keywords:** Myocardial bridge, Coronary artery disease, Myotomy, Coronary artery bypass grafting

## Abstract

**Background:**

The treatment of coronary artery disease combined with severe atherosclerotic stenosis proximal to a left anterior descending artery myocardial bridge (LAD-MB) is still controversial. This study aimed to analyze the outcomes of surgical intervention in patients with severe atherosclerotic stenosis proximal to a LAD-MB.

**Methods:**

We retrospectively reviewed all patients with coronary artery disease combined with severe atherosclerotic stenosis proximal to the LAD-MB. The enrolled criteria were systolic compression of LAD more than or equal to 50% and atherosclerotic stenosis proximal to the LAD-MB more than or equal to 70%. All patients suffered from anginal symptoms refractory to medical therapy. All patients received supra-arterial myotomy and coronary artery bypass grafting (CABG) procedures. Clinical characteristics, intraoperative findings, and postoperative outcomes were evaluated.

**Results:**

Between 2004 and 2021, sixteen patients underwent supra-arterial myotomy and CABG procedure. The compression and length of LAD-MB were 63 ± 17.9% and 25.9 ± 16.3 mm, respectively. Of the 16 patients, one patient had a LAD-MB and proximal coronary stenosis, and 15 patients had LAD-MBs and multivessel lesions. All patients survived and recovered uneventfully without in-hospital mortality or severe complications. The median transfusion amount of red blood cells in the operation was 2 units, and no patients required unplanned reoperation for bleeding. The average length of intensive care unit stay was 2.74 days. Fifteen patients were followed up for 6–146.1 months (mean 45.3 ± 42.9 months). One patient had a recurrence of angina pectoris one year after surgery, and 14 patients had no symptoms of myocardial ischemia during the follow-up period. Significant improvement in symptoms and quality of life using the Seattle Angina Questionnaire assessment was observed in all five categories after surgery (p < 0.01).

**Conclusions:**

Based on the results, supra-arterial myotomy and concomitant bypass surgery may be a better option for the treatment of LAD-MB combined with severe proximal stenosis.

## Introduction

Myocardial bridge (MB) is a congenital coronary artery variant in which a portion of the epicardial coronary arterial segment runs in the myocardium and mainly in the left anterior descending coronary artery (LAD) [1]. It has been characterized by systolic compression of part of an epicardial vessel by a segment of the overlying myocardium. It is one of the most common coronary artery malformations, with a prevalence between 1.5% and 16% assessed by coronary computed tomographic angiography (CCTA) and up to 80% at autopsy [1–3]. Although coronary artery myocardial bridge is usually considered a benign condition, some studies have reported that myocardial bridges are associated with acute coronary syndromes and angina pectoris, fatal arrhythmias, left ventricular dysfunction, and even cardiac sudden death [1]. Our previous studies demonstrated that supera-arterial myotomy is a safe and effective procedure for patients with medication refractory isolated LAD-MB [4–6].

Pathological studies have clearly shown that atherosclerotic changes are present in the intima proximal to the MB [7]. Although the incidence of coronary artery disease combined with atherosclerotic stenosis proximal to the LAD-MB is not reported in the literature, such patients are still found in clinical practice. Unfortunately, there are many contradictions in the choice of the treatment strategy for patients with myocardial bridge and coronary artery disease. In this study, we describe a group of patients who underwent supra-arterial myotomy and coronary artery bypass grafting for severe stenosis proximal to the LAD-MB and summarize the outcomes of their treatment.

## Patients and methods

### Patient population

This retrospective study was approved by the Ethics Committee of First Hospital of Tsinghua University (No. 20210021). We reviewed the hospital's database and analyzed the chart datum, surgical records, and imaging data. The enrolled criteria were systolic coronary diameter compression of LAD-MB more than or equal to 50%, atherosclerotic stenosis proximal to the LAD-MB more than or equal to 70%, the length of LAD-MB was more than 25 mm, or the MB located in the mid-distal segments affecting myocardial perfusion, and all patients suffered from anginal symptoms refractory to medical therapy [8]. This study included 16 patients with coronary artery disease combined with atherosclerotic stenosis proximal to the myocardial bridge of LAD who underwent supra-arterial myotomy and coronary artery bypass grafting at our institute between March 2004 to June 2021.

### Seattle angina questionnaire

The Seattle Angina Questionnaire (SAQ) was administered to patients before undergoing surgical treatment. The questionnaire was repeated at the final postoperative follow-up visit. Pre- and post-operative survey scores are compared. The Seattle Angina Questionnaire is a validated, self-administered questionnaire. It includes five dimensions of functional status: physical limitation due to angina, stability of angina, frequency of angina, satisfaction with treatment, and quality of life [9].

### Surgical procedure

The procedure was performed by median sternotomy, and cardiopulmonary bypass (CPB) was used by cannulating the ascending aorta and right atrium with aortic cross-clamping and performing a cold-blood cardioplegic arrest. For smaller diameter vessels distal to the MBs, myotomy was necessary to make a landing site for the distal anastomosis. Careful proximal dissection was performed with the division of overlying muscle, and avoid damage to the LAD branch or the adjacent diagonal branch vessels, especially avoid right ventricular rupture. Muscle bleeding at the myotomy edges was controlled with electrical cauterization or clips. But for intermuscular arterial or larger venous bleeding, 6/0 prolene sutures (Ethicon, Inc., Somerville, NJ) were used to overrun the bleeding branches on both sides of the myotomy. Harvesting of the left internal mammal artery (LIMA) was the first-choice arterial graft to bypass LAD, and the saphenous vein (SV) was the grafting material for multivessel lesions. A total of 16 patients underwent bypass surgery with in situ LIMA grafting to the distal LAD, 15 of them required simultaneous multivessel revascularization with saphenous veins.

The follow-up data were obtained from our outpatients’ clinic records or by correspondence with referring physicians using telephone and subsequent hospitalization.

### Statistical analysis

Continuous variables were reported as mean ± standard deviation. Non-normally distributed were presented as median (interquartile range [IQR]). Continuous variables were compared using student’s t-test, and the variation of same variable before and after procedure was compared using paired t-test. Statistical significance was defined as a 2-tailed P-value < 0.05. All statistical analysis was performed using IBM SPSS Statistics 22.0 (IBM Corporation, Armonk, NY).

## Results

**Fig. 1:**
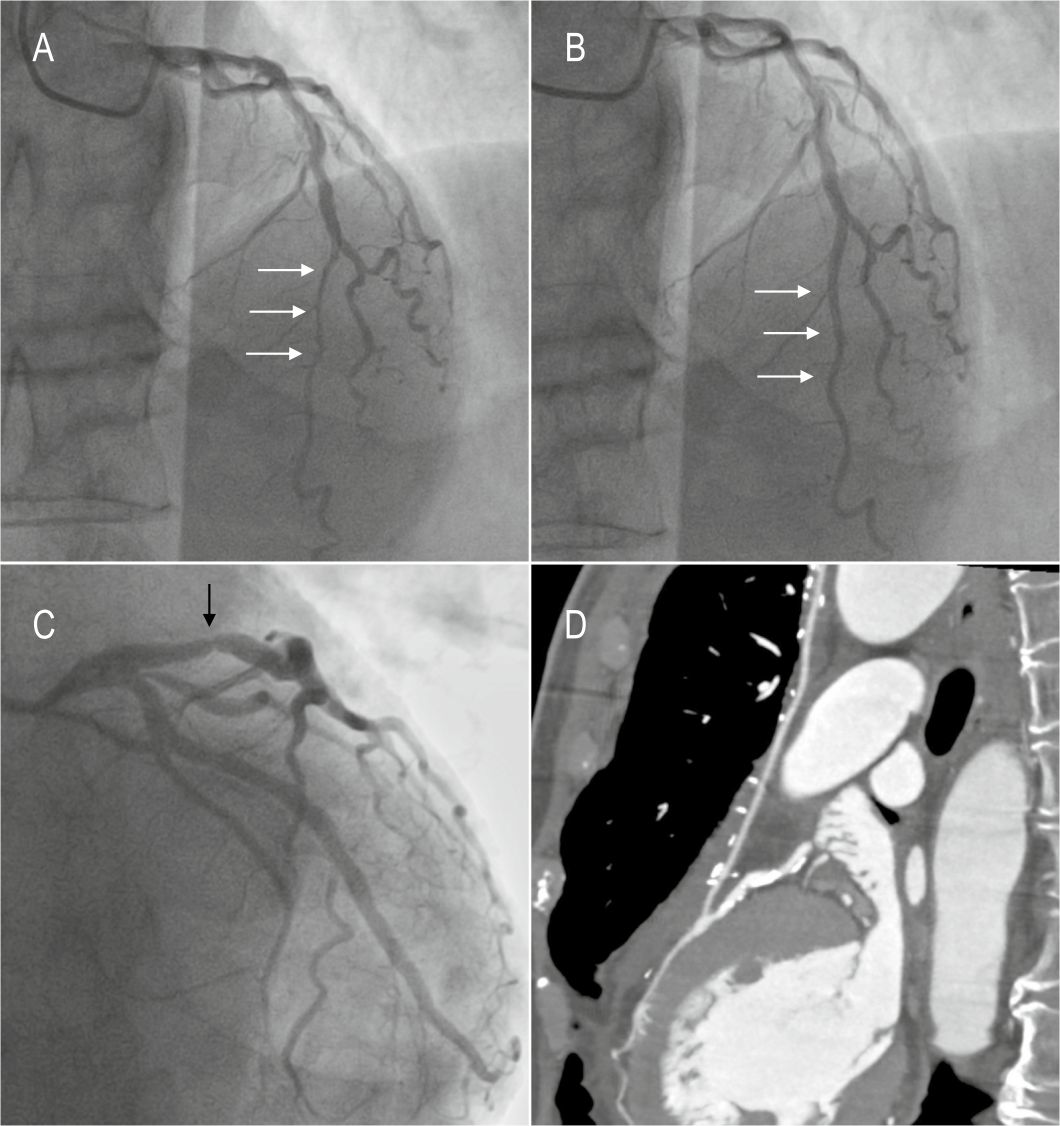
Coronary angiography before surgery and coronary computed tomographic angiography (CCTA) during the follow-up period. Systolic compression of the mid LAD (**A**, white arrow), complete recovery in the same area in diastole (**B**, white arrow). A atherosclerotic stenosis of the proximal segment of the left descending branch (**C**, black arrow). CCTA after surgery demonstrates complete release of MB and graft patency at 6-month follow-up (**D**)

The baseline clinical, echocardiographic and angiographic characteristics are presented in Table 1. Twelve of 16 patients were male, the age ranged from 42 to 75 years (median 60 years). The echocardiography examination confirmed the left ventricular end-diastolic dimension: 49.6 ± 4.7 mm, and the left ventricular ejection fraction: 59.4 ± 6.3%. The compression and the length of LAD-MB were 63 ± 17.9% and 25.9 ± 16.3 mm, respectively. Of the 16 patients, one patient with LAD-MB and proximal coronary stenosis, and 15 patients with LAD-MBs and multivessel lesions.Preoperative coronary angiography showed compression of the LAD-MB during systole (Fig. 1A) and recovery during diastole (Fig. 1B). Atherosclerotic stenosis of the proximal left anterior descending branch artery (Fig. 1C).

**Table 1:** Baseline characteristics

Characteristics	Values
Gender	
Male, n (%)	12 (75)
Female, n (%)	4 (25)
Age, years	60 (51.5, 62)
BSA, m^2^	1.78 ± 0.16
BMI, kg/m^2^	26.1 ± 4.01
Hypertension, n (%)	9 (56.3)
Diabetes mellitus, n (%)	5 (31.2)
Hyperlipidemia, n (%)	7 (43.8)
Peripheral vascular disease, n (%)	0 (0)
Cerebrovascular disease, n (%)	2 (12.5)
Chronic pulmonary disease, n (%)	1 (6.2)
Prior myocardial infarction, n (%)	8 (50)
Atrial fibrillation, n (%)	1 (6.2)
Smoking, n (%)	10 (62.5)
LVEDD, mm	49.6 ± 4.7
LVEF, %	59.4 ± 6.3
Compression of LAD-MB, %	63 ± 17.9
Length of MB, mm	25.9 ± 16.3
Proximal stenosis of LAD, %	89.6 ± 7.2
Medications	
Aspirin, n (%)	9 (56.3)
Beta-blocker, n (%)	10 (62.5)
Calcium-channel blocker, n (%)	1 (6.2)
Nitrates, n (%)	8 (50)

*BSA* body surface area, *BMI* body mass index, *LVEDD* left ventricular end-diastolic diameter, *LVEF* left ventricular ejection fraction

Table 2 lists the operative characteristics and postoperative results. Fifteen patients underwent myotomy and CABG with cardiopulmonary bypass, and one patient received off-pump myotomy and graft surgery. All patients recovered uneventfully without the complications of right ventricular rupture or coronary artery injury during the surgery. The median transfusion amount of red blood cells in the operation was 2 units, and no patients required unplanned reoperation for bleeding. The average length of intensive care unit stay was 2.74 days.

**Table 2:** Operative characteristics and postoperative results

Characteristics	Values
Depth of MB, mm	5 (4, 5)
No. of bypass grafts, n (%)	
1	1 (6.2)
2	5 (31.3)
3	10 (62.5)
In situ ITA, n (%)	16 (100)
Reoperation for bleeding, n (%)	0 (0)
Perioperative myocardial infarction, n (%)	0 (0)
Prolonged ventilation > 72 h, n (%)	0 (0)
Chest wound infection, n (%)	1 (6.3)
Chest drainage on the first day, ml	542.25 ± 217.89
Perioperative RBC transfusion, u	2(0, 4)
Time to extubation, hours	9.5 (6.25, 14.8)
ICU stay, days	2.74 ± 1.6
In-hospital death, n (%)	0 (0)

*MB* myocardial bridge, *CABG* coronary artery bypass grafting, *ITA* internal thoracic artery, *ICU* intensive care unit

Table 3 represents preoperative and final follow-up visit Seattle angina questionnaire (SAQ) score and median change in the score for each category. All five categories (physical limitation due to angina, stability of angina, frequency of angina, satisfaction with treatment, and quality of life) were improved significantly after supra-arterial myotomy and CABG (p < 0.01, Table 3). The follow-up periods ranged from 6 to 146.1 months (mean 45.3 ± 42.9 months), and one patient was lost to follow-up. Of the 15 patients followed up, one patient in this group had a recurrence of angina 1 year after surgery and was found restenosis of the LIMA graft by coronary angiography. The patient's symptoms were reduced by increasing the dose of beta-blockers and calcium antagonists. Fourteen patients had no symptoms of myocardial ischemia, nor major cardiac adverse events during the follow-up periods. Some patients underwent coronary computed tomographic angiography (CCTA) at postoperative review showing complete release of the left anterior descending myocardial bridge and patency of the graft (Fig. 1D).

**Table 3:** Seattle angina questionnaire scores before surgery and at the follow-up period

SAQ scores	Mean ± SD	t	p
Physical limitation due to angina	− 15.84 ± 8.33	− 7.60	< 0.01
Anginal stability	− 71.88 ± 25.61	− 11.22	< 0.01
Anginal frequency	− 36.88 ± 32.81	− 4.50	< 0.01
Treatment satisfaction	− 42.82 ± 19.06	− 8.99	< 0.01
Quality of life	− 30 ± 20.16	− 5.99	< 0.01

## Discussion

Our study demonstrated that in patients with coronary artery disease combined with proximal stenosis to the LAD-MB, supra-arterial myotomy and simultaneous CABG could completely relieve the compression of the myocardial bridge on the tunnel artery and increase blood flow through the graft to the distal branches with satisfactory long-term results.

The coronary myocardial bridge, as a congenital coronary artery malformation, was first described anatomically by Reyman in 1737 [10]. Pathological studies have clearly shown that atherosclerotic changes are present in the intima proximal to the MB [7]. Since the majority of coronary perfusion occurs during the diastolic phase of the cardiac cycle, the effect of systolic compression of the artery on the total effective perfusion of the myocardium should be mild. However, studies have shown that systolic compression of MB results in delayed relaxation of a vessel and leads to impaired diastolic myocardial perfusion [1]. The severity of systolic compression of MB and hemodynamic perturbations in the proximal segment was associated with decreased shear stress and increased blood residence time [11]. A recently study demonstrated that MB located in the middle distal segment of the LAD is independently associated with coronary stenosis proximal to the MB, but the prevalence of coronary artery disease combined with LAD-MB are unclear [12]. For the dynamic bridge compression averaging 55% diameter stenosis, the main artery downstream has adequate coronary flow reserve and fractional flow reserve (FFR) but the isolated septal artery may be ischemic due to septal “branch steal” [13]. In clinical cases, there is a large number of patients with atherosclerotic stenosis proximal to LAD-MB combined with multiple coronary artery lesions. For these patients, whether complete supra-arterial myotomy is performed in conjunction with CABG has not been reported in the literature. In this study, although postoperative coronary angiography or CCTA was not performed in all patients, no serious adverse cardiac events such as myocardial infarction, arrhythmia, or sudden cardiac death occurred in the patients during long-term follow-up. We suggest that it may be useful in relieving severe myocardial bridge compression caused by the tunnel artery and branches steal, thereby increasing diastolic blood supply and reducing myocardial ischemia.

Coronary artery bypass grafting can increase blood flow through the graft to the distal branches for patients with severe atherosclerotic lesions proximal to MBs. However, for the patients with vessel diameter of less than 1.5 mm distal to the myocardial bridge, it is difficult to select a suitable anastomosis site for patients with an extensive MB without performing an MB myotomy [14, 15]. In particular, for diffuse myocardial bridges located in the middle and distal segments of the anterior descending branches with a distal caliber of less than 1.5 mm, a myotomy is necessary. The key point for this procedure is to find the exact location of MB by angiographic findings and dissect the cardiac muscle carefully and completely. Concerning the choice of treatment strategy, there is no literature on whether to completely or partially release the myocardial bridge in patients with atherosclerotic stenosis proximal to the LAD-MB. Although postoperative intravascular ultrasound (IVUS) was not performed in this study, based on the prior literature [13], we believe that complete release of the compression caused by the myocardial bridge increases anterior wall and septal perfusion and reduces myocardial ischemia. In the case of diffuse myocardial bridges, we usually use a retrograde approach to select the target vessel site. It is of concern that avoiding right ventricular rupture and coronary artery injury is a key technique for myotomy. Our experience is that surgery under cardiac arrest with extracorporeal circulation is a safe and effective method for the patients with diffuse myocardial bridges.

In this small cohort of patients, we used the Seattle Angina Questionnaire (SAQ) score to compare quality of life before and after CABG and myotomy. We observed a significant less physical limitation, lower angina frequency and a significant symptomatic improvement in across all five dimensions of the SAQ after surgery. Bianco F and colleagues analyzed the echocardiographic changes and quality of life after surgical unroofing of myocardial bridges and demonstrated that MB unroofing surgery could provide benefits in terms of quality of life and left ventricular global longitudinal strain improvement compared with one year of optimal medical therapy [16]. Maeda analyzed 13 pediatric myocardial bridge patients who underwent surgical unroofing of MB and all patients had significant improvement in symptoms and quality of life using the SAQ score at postoperative follow-up [17]. Overall, surgical unroofing of myocardial bridges can relieve the compression of the tunnel artery, relieve myocardial ischemia, and improve the patients' quality of life after surgery.

Limitations of this study: first, it is a single-institute retrospective, observational study with a small sample size, which would affect the generalizability of the findings. Multiple centers, large sample, randomized controlled studies are needed for final study conclusions. Second, only some patients in this study underwent postoperative coronary angiography or CCTA, and most patients were followed up with symptom questionnaires, making evaluation difficult to achieve accuracy.

## Conclusions

For patients with coronary artery disease combined with extensive LAD-MB and proximal stenosis, performing a myotomy is necessary to make a landing site for the distal anastomosis and provide better coronary perfusion to areas distal to the coronary artery. Supra-arterial myotomy concomitant bypass surgery may be a better option for the treatment of LAD-MB combined with severe proximal stenosis.

## Data Availability

The datasets used and/or analyzed during the current study are available from the corresponding author on reasonable request.
